# A Morphological Study of Retinal Changes in Unilateral Amblyopia Using Optical Coherence Tomography Image Segmentation

**DOI:** 10.1371/journal.pone.0088363

**Published:** 2014-02-06

**Authors:** Andrea Szigeti, Erika Tátrai, Anna Szamosi, Péter Vargha, Zoltán Zsolt Nagy, János Németh, Delia Cabrera DeBuc, Gábor Márk Somfai

**Affiliations:** 1 Department of Ophthalmology, Semmelweis University, Budapest, Hungary; 2 Cardiovascular Centre, Semmelweis University, Budapest, Hungary; 3 Bascom Palmer Eye Institute, University of Miami Miller School of Medicine, Miami, Florida, United States of America; Medical University of South Carolina, United States of America

## Abstract

**Objective:**

The purpose of this study was to evaluate the possible structural changes of the macula in patients with unilateral amblyopia using optical coherence tomography (OCT) image segmentation.

**Patients and Methods:**

38 consecutive patients (16 male; mean age 32.4±17.6 years; range 6–67 years) with unilateral amblyopia were involved in this study. OCT examinations were performed with a time-domain OCT device, and a custom-built OCT image analysis software (OCTRIMA) was used for OCT image segmentation. The axial length (AL) was measured by a LenStar LS 900 device. Macular layer thickness, AL and manifest spherical equivalent refraction (MRSE) of the amblyopic eye were compared to that of the fellow eye. We studied if the type of amblyopia (strabismus without anisometropia, anisometropia without strabismus, strabismus with anisometropia) had any influence on macular layer thickness values.

**Results:**

There was significant difference between the amblyopic and fellow eyes in MRSE and AL in all subgroups. Comparing the amblyopic and fellow eyes, we found a statistically significant difference only in the thickness of the outer nuclear layer in the central region using linear mixed model analysis keeping AL and age under control (p = 0.032). There was no significant difference in interocular difference in the thickness of any macular layers between the subgroups with one-way between-groups ANCOVA while statistically controlling for interocular difference in AL and age.

**Conclusions:**

According to our results there are subtle changes in amblyopic eyes affecting the outer nuclear layer of the fovea suggesting the possible involvement of the photoreceptors. However, further studies are warranted to support this hypothesis.

## Introduction

Amblyopia remains an important cause of low visual acuity, affecting 2% to 6% of the general population [1]–[5]. Unilateral amblyopia is defined as reduced best-corrected visual acuity (BCVA) secondary to an abnormal visual experience during the critical period of visual development. Classic causes include strabismus, anisometropia, form deprivation or a combination of these factors [6].

The neural sites that are influenced by visual deprivation are still under investigation. Nevertheless, it has been reported by several studies in humans [7], [8] and also in animal species [9]–[12] that visual deprivation has an effect on the cell growth in the lateral geniculate body that receives input from the amblyopic eye and on the shift in the dominance pattern in the visual cortex [13], Banko et al revealed that latencies of the event-related potential components increased and were more variable in the amblyopic eye compared to the fellow eye [14], although the initial neural site of the visual deficit in this condition is still under investigation.

Evidences for direct retinal changes in amblyopic eyes are still inconclusive and controversial [15]–[17], although electroretinograms elicited by patterned stimuli in humans with various types of amblyopia were found to be significantly reduced [18], [19]. Studies using optical coherence tomography (OCT) imaging of the retina have produced discordant results, some investigators have found an increased peripapillary retinal nerve fiber layer (cpRNFL) [20]–[22] or/and macular thickness [20], [23]–[26] in amblyopic eyes, whereas others have found no significant differences between amblyopic and healthy eyes [27]–[32].

Yen et al. hypothesized that the normal postnatal reduction (apoptosis) of retinal ganglion cells is arrested in amblyopia and predicted that this would cause increased cpRNFL thickness [21]. If this does indeed occur, it is likely that the arrest of normal postnatal changes would result not only in increased RNFL thickness but also would affect the normal maturation of the macula, including movement of Henle’s fibers away from the foveola and a decrease in foveal cone diameter. This would result in increased foveal thickness [21]. Furthermore, because of the reduced apoptosis of retinal ganglion cells, the thickness of the ganglion cell layer in the macula would also be increased.

The purpose of this study was to evaluate the structural changes of the macula in patients with unilateral amblyopia using OCT image segmentation.

## Materials and Methods

### Subjects

This prospective study was performed at the Department of Ophthalmology, Semmelweis University, Budapest, Hungary, on 38 consecutive Caucasian patients (16 male; mean age 32.4±17.6 years; range 6–67 years) with unilateral amblyopia who presented at the Orthoptics Outpatient Clinic of the department for examination. Only amblyopic eyes with BCVA of 20/30 or worse were included in the study. The BCVA of the fellow eye was 20/20 in each patient.

Patients with a history of intraocular surgery, retinal or neurological disease (e. g. multiple sclerosis), optic nerve disease including glaucoma, nystagmus, laser treatment or any ocular media opacities including cataract were excluded from the study. All participants were treated in accordance with the tenets of the Declaration of Helsinki. Institutional Review Board approval was obtained for all study protocols (Semmelweis University Regional and Institutional Committee of Sciences and Research Ethics). Written informed consent was obtained from all participants in this study. In case of subjects under 18 years of age, written informed consent was obtained from a parent or a legal guardian.

In order to study the effect of the type of amblyopia on interocular difference (IOD) in the different macular layers, patients were divided into 3 study groups: strabismic patients without anisometropia (Group S), anisometropic patients without strabismus (Group A) and combined amblyopic patients with strabismus and anisometropia (Group AS). Anisometropia was defined as an IOD of at least 1.5 diopters of the manifest spherical equivalent refraction (MRSE). For the group descriptions see Table 1.

**Table 1:** Descriptive statistics and distribution of study participants. (SD: standard deviation).

Amblyopic groups	Number	Gender (m/f)	Age (years) mean ± SD (range)	Characteristic	Number
**Group S**	17 (44.7%)	6/11	31.2±14.9(8–56)	Esotropia	12
				Exotropia	5
**Group A**	11 (28.9%)	5/6	39.9±24.2(6–67)	spherical hyperopia	9
				spherical myopia	2
**Group AS**	10 (26.3%)	5/5	37.9±18.0(12–67)	spherical hyperopia+ esotropia	5
				spherical hyperopia+ exotropia	5
				spherical myopia+esotropia	0
				spherical myopia +exotropia	0
**Total**	38 (100%)	16/22	32.4±17.6(6–67)		

(Group S: strabismic patients without anisometropia; Group A: anisometropic patients without strabismus; Group AS: combined amblyopic patients with strabismus and anisometropia).

All patients underwent a comprehensive eye examination, including BCVA (measured with Snellen chart adjusted at 5 m, converted to logMAR values for analysis), cover and cover-uncover test, duction and version testing, measurement of the intraocular pressure, slit-lamp and indirect fundus examination after pupil dilation and determining manifest refraction and cycloplegic refraction after pupil dilation with 3 times cyclopentolate 1% drops.

Spherical equivalent was defined as the spherical power plus half of the minus cylinder power (sphere+ ½ cylinder).

The axial length (AL) of the eyes was measured using a LenStar LS 900 device (LS 900® Haag-Streit AG, Koeniz, Switzerland).

### Optical Coherence Tomography

OCT examinations were performed on each eye using a time-domain (TD) Stratus OCT device (Carl Zeiss Meditec, Dublin, CA), by the same operator through dilated pupils at least 5 mm in diameter. “Fast RNFL map protocol” consisting of three circular scans with diameters of 3.4 mm centered on the optic disc was performed along with the “Macular Thickness Map” protocol consisting of six radial scan lines centered on the fovea, each having a 6 mm transverse length. In order to obtain the best image quality, focusing and optimization settings were controlled and scans were accepted only if the signal strength (SS) was >6 (preferably 9–10). Scans with foveal decentration [i.e., with center point thickness standard deviation (SD) >10%] were repeated. Patients who had poor fixation cooperation due to poor vision or low age were excluded from the study.

**Figure 1:**
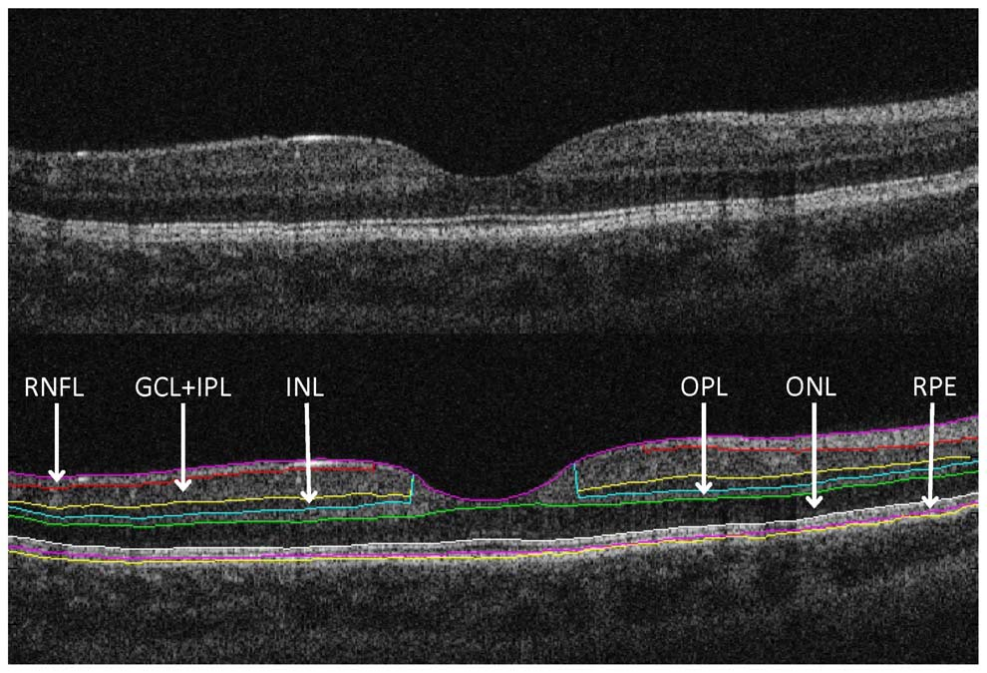
Macular image segmentation using OCTRIMA software. Top: the image of a healthy macula scanned by Stratus OCT. Bottom: the same OCT scan processed using OCTRIMA software. Abbreviations: GCL+IPL, ganglion cell layer and inner plexiform layer complex; INL, inner nuclear layer; ONL, outer nuclear layer; OPL, outer plexiform layer; RNFL, retinal nerve fiber layer; RPE, retinal pigment epithelium.

The mean overall cpRNFL thickness values measured by the built-in software of Stratus OCT were recorded for each eye. In order to assess the thickness of intraretinal layers, the raw data of all 6 OCT images were exported from the device and processed using a custom-built image analysis software (OCTRIMA) [33], [34]. The OCTRIMA software’s methodology includes the removal of speckle noise followed by the automatic detection of the boundaries of the cellular layers of the retina. Manual correction of the boundary detection is enabled if segmentation errors are present. The repeatability and reproducibility of the thickness measurements was assessed earlier and was found to be very good [35], [36]. All the scans were segmented by the same blinded operator to reduce the possible bias caused by different operators and unblinded analysis. (Figure 1).

The thickness values for the RNFL, ganglion cell layer and inner plexiform layer complex (GCL+IPL), inner nuclear layer (INL), outer plexiform layer (OPL), outer nuclear layer (ONL), retinal pigment epithelium (RPE) and the total retina measured by OCTRIMA were recorded for each eye in the nine regions of the macula defined by the Early Treatment Diabetic Retinopathy Study (ETDRS) Group. [37] The distribution of the regions is the following: 1 central, 2 inner superior, 3 inner nasal, 4 inner inferior, 5 inner temporal, 6 outer superior, 7 outer nasal, 8 outer inferior, 9 outer temporal. The thickness of the ganglion cell complex (GCC) composed by the RNFL and GCL+IPL consisting of distal and proximal parts of the retinal ganglion cells was also determined to determine the integrity of the ganglion cells.

In order to assess the intraretinal layers in various eccentricities from the fovea, the mean thickness of the layers for the central (R1), pericentral (R2–R5) and peripheral (R6–R9) ETDRS regions of the macula were calculated. Since the number of sampling points is different at the central, pericentral and peripheral regions because of the radial spoke pattern used in the scanning protocol of Stratus OCT, a weighted mean thickness was calculated for the total macula instead of averaging retinal thickness results in the 9 macular regions [38]. The weighted mean thickness was generated using the following equation:
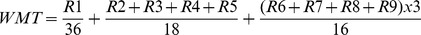



### Statistical Analysis

The magnitude of anisometropia and IOD in BCVA, AL and macular thickness were obtained by subtracting the value obtained in the fellow eye from the value obtained in the amblyopic eye.

The distributions of MRSE, BCVA, AL and macular thickness were confirmed as normally distributed by the Shapiro-Wilks W and Kolmogorov-Smirnov tests and therefore macular thickness, AL and MRSE of the amblyopic eye was compared to that of the fellow eye using the paired t-test.

In order to prevent multicollinearity we examined the Pearson correlation between the independent variables (AL, BCVA, MRSE) before the multiple regression analysis. The IOD in BCVA did not correlate either with IOD in AL or with IOD in MRSE (Pearson correlation IOD in BCVA vs. AL r = 0.217 p = 0.218 n = 34; Pearson correlation IOD in BCVA vs. MRSE r = 0.015 p = 0.929 n = 38), while IOD in MRSE (i.e. anisometropia) correlated with the IOD in AL (Pearson correlation r = −0.602, p<0.0001, n = 34). Thus, we excluded anisometropia as an independent variable from the multiple regression model.

Multiple regression was performed between interocular difference in macular layers as dependent variable and IOD in AL and BCVA, which showed statistically significant correlation between IOD in the thickness of most retinal layers and IOD in AL. (p<0.05), while IOD in BCVA did not correlate with IOD in most retinal layer thickness data. (p>0.05) (see Table 2).

**Table 2:** Multiple regression analysis between interocular difference (IOD) in macular layers as dependent variable and IOD in axial length (AL) and best-corrected visual acuity (BCVA).

IOD in retinal layers	Model adjustedR square	Independent variable			
		IOD in AL		IOD in BCVA	
		beta	p	beta	p
**Mean thickness**					
RNFL	0.287	0.309	**0.049**	0.422	**0.009**
GCL+IPL	0.094	−0.376	**0.034**	−0.038	0.822
GCC	0.040	−0.134	0.448	0.314	0.082
INL	0.127	−0.423	**0.016**	0.187	0.270
OPL	0.112	−0.298	0.086	0.349	**0.046**
ONL	0.377	−0.549	**0.000**	−0.238	0.101
RPE	−0.018	−0.203	0.267	−0.023	0.900
*total macula*	0.158	−0.461	**0.008**	0.181	0.279
**Central region**					
ONL	0.146	−0.364	**0.035**	0.345	**0.044**
RPE	0.094	−0.260	0.135	−0.234	0.177
*total macula*	0.078	0.197	0.258	0.268	0.127
**Pericentral region**					
RNFL	0.012	0.182	0.312	0.162	0.369
GCL+IPL	0.090	0.364	**0.041**	0.059	0.732
GCC	0.160	0.404	**0.019**	0.147	0.375
INL	0.058	0.339	0.059	0.004	0.980
OPL	0.020	−0.064	0.721	0.289	0.112
ONL	0.343	−0.489	**0.002**	−0.288	0.055
RPE	−0.020	−0.168	0.358	−0.087	0.631
*total macula*	−0.057	−0.060	0.746	0.074	0.690
**Peripheral region**					
RNFL	0.322	0.311	**0.042**	0.453	**0.004**
GCL+IPL	0.204	−0.487	**0.005**	−0.056	0.728
GCC	0.084	−0.309	0.080	0.286	0.103
INL	0.291	−0.586	**0.000**	0.209	0.173
OPL	0.162	−0.398	**0.021**	0.335	0.049
ONL	0.356	−0.559	**0.000**	−0.191	0.193
RPE	−0.024	−0.199	0.278	0.023	0.901
*total macula*	0.247	−0.550	**0.001**	0.181	0.250
**cpRNFL**	0.029	−0.199	0.284	0.269	0.152

Adjusted R square, which indicates the strength of the model, standardised regression coefficients (beta) and p values.

GCL+IPL: ganglion cell layer and inner plexiform layer complex; GCC: ganglion cell complex; INL: inner nuclear layer, OPL: outer plexiform layer; ONL: outer nuclear layer; RPE: retinal pigment epithelium.

Linear correlation was calculated between intraretinal layer thickness data and age in the control eyes which revealed significant correlation for the ONL, RPE layers and the total macula in the central region (see Table 3), therefore we controlled our further analyses for age as well.

**Table 3:** Linear correlation for intraretinal layer thickness data and age in the non-amblyopic eyes.

Control eyes	Pearson correlation	p
**mean thickness**		
ONL	−0.349	**0.032**
RPE	0.615	**0.000**
**central region**		
RPE	0.355	**0.029**
*total macula*	0.416	**0.009**
**pericentral region**		
RPE	0.594	**0.000**
**peripheral region**		
ONL	−0.385	**0.017**
RPE	0.578	**0.000**

Only layers with a statistically significant correlation are shown.

One-way between-groups ANCOVA was used to test if the type of amblyopia (Group S, A, AS) had influence on the IOD in macular layer thickness between amblyopic and fellow eyes. Because of the above lack of correlation between IOD in BCVA and IOD in retinal layer thickness, the test was controlled for IOD in AL and age. In order to compare macular thickness data linear mixed model was used, keeping AL and age under control.

Statistical analyses were performed using SPSS software program (Statistical Package for Social Sciences, SPSS version 16.0; SPSS Inc., Chicago, IL, USA). A p value of <0.05 was considered statistically significant.

## Results

A total of 38 patients with unilateral amblyopia were enrolled in this study. Altogether 17 patients (44.7%) had strabismic amblyopia, 11 patients (28.9%) had anisometropic amblyopia, and 10 patients (26.3%) had combined amblyopia (strabismus and anisometropia). Of the patients with anisometropia, spherical hyperopia was noted in 9 cases and myopia was present in 2 cases. Among patients with strabismus, esotropia was noted in 12 cases with a mean of 22±14 (range: 6–40) prism diopters (PD) deviation, and exotropia was noted in 5 cases with a mean of 35±12 (range: 14–40) PD deviations. In the mixed cases (Group AS), there were 5 cases in which spherical anisometropic hyperopia and exotropia (mean deviation: 28±10 PD, range: 14–40 PD) were concomitantly present, and there were 5 cases with anisometropic hyperopia combined with exotropia (mean deviation: 30±10 PD, range: 20–40 PD). (Table 1) There were 12 right and 26 left amblyopic eyes.

The mean BCVA was +0.69±0.39 logMAR in all amblyopic eyes involved in the study and 0.00 in all fellow eyes. In the S, A and AS subgroups the mean BCVAs were +0.67±0.44 logMAR; +0.75±0.34 logMAR; and +0.64±0.38 logMAR, respectively.

The mean MRSE in the amblyopic eyes was +2.97±2.39 diopters (D) and in the normal fellow eyes was +1.65±1.87 D. The mean AL was 22.51±1.08 mm in the amblyopic eyes and 22.96±1.03 mm in the fellow eyes. Table 4 shows the MRSE and AL values in the different subgroups. There was statistically significant difference between the amblyopic and fellow eye in MRSE and AL in all subgroups. Two patients with strabismic amblyopia and two patients with anisometropic amblyopia did not undergo axial length measurements due to technical reasons.

**Table 4:** Manifest refraction in spherical equivalent (MRSE) and axial length in the study subjects compared by paired t-test.

MRSE (D)	N	Amblyopic eyemean ± SD(range)	Fellow eyemean ± SD(range)	P values
All amblyopicpatients	38	2.97±2.39(−2.75–8.88)	1.65±1.83(−1.38–6.88)	**0.000**
Group S	17	2.31±1.96(−1.00–7.50)	1.88±2.03(−1.38–6.88)	**0.007**
Group A	11	2.74±3.06(−2.75–8.88)	0.99±1.11(−0.5–3.13)	**0.001**
Group AS	10	4.36±1.78(1.5–7.5)	1.98±2.06(−0.5–5.5)	**0.000**
**Axial length** **(mm)**	**N**	**Amblyopic eye** **mean ± SD** **(range)**	**Fellow eye** **mean ± SD** **(range)**	**P values**
All amblyopicpatients	34	22.51±1.08(20.78–25.68)	22.96±1.03(20.95–25.08)	**0.001**
Group S	15	22.49±0.88(21.02–23.94)	22.72±0.96(21.38 to 24.38)	**0.028**
Group A	9	22.86±1.69(20.78–25.68)	23.11±1.35(20.95–25.08)	**0.002**
Group AS	10	22.21±0.58(21.34 23.08)	23.17±0.82(22.1 24.36)	**0.003**

Note that two patients with strabismic amblyopia and two patients with anisometropic amblyopia did not undergo axial length measurements due to technical reasons.

The mean macular layer thickness and cpRNFL thickness measurements are shown in Table 5. Comparing the amblyopic and fellow eyes without including any other parameters in the test there was a slight statistically significant difference in the GCC layer in the pericentral region and in the OPL layer calculated for the total macula and measured in the peripheral region (paired t test; p = 0.018; p = 0.014). However, after implementing a linear mixed model keeping AL and age under control, the above statistically significant differences disappeared while significant difference was present only in the ONL layer in the central region (p = 0.032). Using the same model and implementing an ideal AL of 22.73 mm and an age of 32.79 years, the estimated ONL thickness would be 120.69 µm in the amblyopic eye and 120.28 µm in the fellow eye.

**Table 5:** Macular layer thickness values and cpRNFL (µm) data in the study eyes with p values for the comparisons between amblyopic and fellow eyes by two different methods.

	Amblyopic eye (n = 38)Macular layer thickness (µm)mean ± SD	Fellow eye (n = 38)Macular layer thickness (µm)mean ± SD	P valuesPaired t test(n = 38)	P valuesMixed model (controlled age and AL) n = 34
**Mean thickness**				
RNFL	36.53±3.14	36.95±2.78	0.265	0.583
GCL+IPL	71.3±5.89	71.36±5.12	0.912	0.860
GCC	107.83±6.9	108.31±6.75	0.361	0.772
INL	34.51±2.32	34.48±1.76	0.928	0.978
OPL	33.43±1.65	32.87±1.43	**0.032**	0.844
ONL	81.98±6.93	81.92±6.38	0.898	0.083
RPE	12.3±1.34	12.48±1.32	0.332	0.205
*Total retina*	296.39±13.74	296.05±12.28	0.766	0.379
**Central Region**				
ONL	120.02±8.69	119.78±10.38	0.836	**0.032**
RPE	14.98±1.61	14.94±1.45	0.861	0.410
*Total retina*	242.22±19.68	241.34±22.35	0.631	0.985
**Pericentral Region**				
RNFL	23.29±3.15	23.86±2.52	0.191	0.266
GCL+IPL	93.95±6.49	95.02±6.26	0.079	0.360
GCC	117.23±8.02	118.88±7.62	**0.018**	0.873
INL	38.67±3.22	39.01±2.41	0.277	0.936
OPL	38.84±2.66	38.37±2.33	0.320	0.471
ONL	90.92±8.42	91.03±8.73	0.892	0.328
RPE	12.54±1.71	12.49±1.72	0.842	0.284
*Total retina*	323.6±15.26	324.64±13.77	0.272	0.341
**Peripheral Region**				
RNFL	41.81±3.55	42.2±3.35	0.365	0.805
GCL+IPL	67.23±6.6	66.99±5.51	0.863	0.932
GCC	109.04±7.46	109.19±7.28	0.819	0.846
INL	34.55±2.49	34.42±1.97	0.645	0.957
OPL	33.07±1.61	32.46±1.47	**0.014**	0.481
ONL	77.92±6.73	77.93±6.1	0.989	0.242
RPE	12.12±1.31	12.40±1.31	0.167	0.247
*Total retina*	290.29±14.45	289.61±12.63	0.601	0.413
**CpRNFL**	106.10±10.37	104.43±8.74	0.182	0.098

Data are presented as mean ± SD. Comparisons were performed by paired t-test and a linear mixed model keeping axial length and age under control.

We studied whether the type of amblyopia had any influence on the IOD in macular layers between amblyopic and fellow eye with one-way between-groups ANCOVA while statistically controlling for IOD in AL and age. There was no significant difference in IOD in macular layers and cpRNFL between Group S, A and AS.

## Discussion

In this study we assessed the retinal structural changes of the macula in patients with unilateral amblyopia using OCT imaging and image segmentation methodology.

Amblyopia occurs during the period when the neuronal network between the retina and the cerebral cortex is developing and maturing. The neural sites that are influenced by visual deprivation are still under investigation. However, some animal studies demonstrated abnormal findings in retinal microstructures, including degeneration of retinal ganglion cells [39], [40], decreased nucleolar volume and cytoplasmic cross-sectional area of RGCs [16], an increased number of amacrine synapses in the IPL [41], [42], a reduction in the number of bipolar synapses in the IPL [41], thinning of the IPL [16], [43], and a decrease in the density of Müller fibres [43].

Evidence for direct retinal changes in amblyopic eyes remain inconclusive and controversial. Yen et al. hypothesized that amblyopia may affect the postnatal maturation of the retina, including the postnatal reduction of retinal ganglion cells, which would lead to a measurable increase in the thickness of the retinal nerve fiber layer in amblyopic eyes [21]. If this indeed occur, it is likely that the arrest of normal postnatal changes would result not only in increased RNFL thickness but also would affect the normal maturation of the macula, including movement of Henle’s fibers away from the foveola and a decrease in foveal cone diameter, and would result in increased foveal thickness [21]. According to this assumption and the above mentioned animal studies we could reasonably hypothesize that some anatomic rearrangement could be present in the retina.

A small number of previous studies that aimed the assessment of retinal structural changes in amblyopia has been reported. Enoch et al. were the first of many authors to suggest a specific cause for an organic anomaly affecting the retina in amblyopia [44]. More recently, using a third-generation nerve fiber analyzer (GDx, Laser Diagnostic Technologies, San Diego, CA), Colen et al. measured RNFL thickness in strabismic amblyopia and reported no significant difference between amblyopic and sound eyes [45]. In 2005, Altintas et al. carried out OCT examination on 14 unilateral strabismic amblyopic patients and no difference was seen in macular and cpRNFL thickness or macular volume [27]. Kee et al. enrolled 26 unilateral amblyopic children (6 strabismic, 15 anisometropic, 5 combined amblyopes), and found no difference in cpRNFL in any of examined 4 quadrants (superior, inferior, nasal, temporal) and foveal thickness between neither the amblyopic eye and fellow eye, nor between values of these amblyopic patients and 42 normal control children using time-domain OCT [29]. However, they found statistically significant difference in mean thickness values of the fovea and the retinal nerve fiber layer of the amblyopic eyes of the children with anisometropic amblyopia (n = 15) and strabismic amblyopia (n = 6) (146.5 vs. 173.1 µm p = 0.046, cpRNFL 112.9 vs. 92.8 µm p = 0.034) [29]. They did not measure the AL, and in the anisometropic group 10 of 15 children were myop.

Repka et al. completed studies in 2006 and 2009 evaluating 17 patients aged 5–28 years and subsequently 37 amblyopic children and found no difference in cpRNFL thickness between amblyopic and sound eyes using TD-OCT [30], [31]. Similarly, in 2011, Walker et al. investigated 30 adults (mean age: 56 years) with amblyopia (using Cirrus HD-OCT) and found no statistically significant difference in RNFL thickness of any peripapillary quadrants and macular thickness in any anatomical location [32].

In 2004, Yen et al. used 2nd generation optical coherence tomography to measure cpRNFL in 38 patients (mean age 26.4, range 6–75 years) with unilateral amblyopia (strabismic and refractive amblyopia) and found no significant difference between strabismic amblyopic and normal eyes [21]. However, the cpRNFL was significantly thicker in eyes with refractive amblyopia compared with the fellow eye and the differences were significant in the multivariate regression analysis as well with adjustment for AL, SE, age and sex [21]. Yoon et al had similar findings in a study of 31 hyperopic anisometropic children regarding cpRNFL thickness (115.2 vs. 109.6 µm, p = 0.019) but found no difference in mean macular retinal thickness (252.5 vs. 249.7 µm) [22]. They did not measure the AL.

In the Sydney Childhood Eye Study, Huynh et al. tested 48 unilateral amblyopes (17 strabismic, 19 hyperopic anisometropia) and reported that amblyopic eyes had slightly greater foveal minimum thickness than the normal fellow eye (by 5.0 µm) and the right eyes of non-amblyopic children (by 10 µm) [25]. This difference was more pronounced in 6-year old children (6.9 µm) than 12–year old children (4.2 µm) [25]. The IOD in foveal minimum thickness was greater in children who did not receive any treatment for unilateral amblyopia [25]. Foveal minimum thickness remained significantly greater in amblyopic than non-amblyopic eyes, after adjusting for amblyopia severity and IOD in AL (p = 0.01). In their study the inner macular ring was significantly thinner in amblyopic children, and there were no significant differences in outer macular ring thicknesses, central and total macular volume or in cpRNFL [25].

In 2011, Alotaibi et al. evaluated 93 unilateral amblyopic eyes (36 strabismic, 33 anisometropic, 24 combined) and found significantly thicker RNFL (259.3 vs. 255.6 µm, p<0.0001) in the overall amblyopic group, and no significant difference in macular and foveal thickness [20]. There was slightly higher macular and foveal thickness only in the anisometropic amblyopic group (macular thickness: 256.76 vs. 246.61 µm p = 0.050; foveal thickness: 187.12 vs. 177.61 µm p = 0.039) [20]. However, they did not measure the AL either.

In the studies by Dickmann et al. a significant difference between the amblyopic and the fellow eye was found in mean macular thickness only in the strabismic amblyopic group, and there was no difference for the refractive amblyopic group, similarly to the cpRNFL in any of the amblyopic groups [23], [24]. Alotaibi and Dickmann suggested based on their findings that amblyopia of different etiologies is associated with the loss of different neural cells [20], [23], [24]. Later, in 2012, Dickmann evaluated 15 strabismic (esotropic) and 15 anisometropic amblyopic patients, and found no intereye differences in cpRNFL, macular thickness and foveal volume in neither group using spectral-domain (SD) OCT [28].

In 2011, Pang et al. investigated 31 myopic children with unilateral amblyopia [26]. The refractive error in spherical equivalent in the amblyopic eyes was −10.79±3.40 diopters and in the normal fellow eyes was −1.67±2.90 D. The mean magnitude of anisometropia was 9.12±3.53 D, ranging from 3.63 to 17.50 D. They found a statistically significant difference in macular thickness between amblyopic and fellow eyes, with amblyopic eyes having greater foveal thickness but reduced inner and outer macular thickness [26]. No statistically significant differences were indentified in the macular thicknesses between subgroups (purely myopic anisometropia n = 24, combined mechanism amblyopia n = 7). IOD in AL (measured AL with A-scan ultrasound biometry) showed a moderate correlation with the nasal, superior and temporal outer macular thickness [26].

To the best of our knowledge, there are three recent studies that employed some form of OCT image segmentation in amblyopia so far. Al-Haddad et al used one single horizontal SD-OCT scan for the manual segmentation of six layers of the central 1000 µm diameter area and found an increase in the INL and a decrease in the ONL in the temporal area in amblyopic eyes compared to the fellow eyes, while the mean foveal thickness was increased in amblyopic eyes (228.56±20.2 vs 221.7±15.3 µm) [46]. Tugcu et al. used the built-in analysis option of the RTVue OCT platform to measure the thickness of the GCC and found an increase in strabismic amblyopia (99.29 vs. 103.08 µm, p = 0.019, amblyopic vs. nonamblopic eye) while there was no such difference for the anisometropic or combined subgroups [19]. Park et al. enrolled 20 unilateral amblyopic children (16 strabismus, 2 anisoastigmatism, 2 unilateral ptosis) with a mean age of 9.0±4.03 (4–19 years) and examined horizontal and vertical spectral-domain OCT scans through the fovea [47]. Thickness values were measured at the foveal centre and in 500 and 1500 µm distance from the foveal centre in all 4 quadrants (superior, inferior, nasal, temporal). The thickness of each retinal layer (GCL+IPL, INL, OPL, ONL, IS, OS, RPE) was measured manually using the calipers provided with the SD-OCT instrument [47]. They found significantly decreased thickness in the thickness of the GCL+IPL at all four nasal and temporal macular locations and at the outer superior and inferior locations [47]. The ONL was thinner at the inner and outer temporal locations and thicker at the inner and outer superior and inner nasal locations. The NFL and OPL were thicker in the amblyopic eyes than in the fellow eyes in some areas and thinner in other areas [47]. It should be noted that none of these studies corrected their results for either age or axial length.

In the present study we used OCT image segmentation methodology involving the entire macular area, extracting seven retinal layers. We found that subtle changes may be present in the retina in unilateral amblyopia. As there was evidence showing the confounding effect of AL and age we used state-of-the-art statistical methodology in order to keep these variables under control. As most of the similar previous studies in the field were using basic comparisons, we also performed such analyses. Interestingly, the basic pairwise comparisons were indicating significant changes in the GCC layer in the pericentral region and in the OPL layer calculated for the total macula and measured in the peripheral region. However, after applying rigorous statistical methodology in order to account for the effects of AL and age, these differences disappeared, while a significant difference was revealed for the central (foveal) ONL. This implies that the photoreceptors and not the ganglion cells could be affected by amblyopia, this being the opposite of what was earlier speculated.

Previous studies reported that there was a correlation between retinal thickness and AL, age, or even race in healthy eyes [48]–[52] and that AL may have an effect on the thickness of the intraretinal layers in the macula [53]. Song et al. found that axial length correlated negatively with average outer macular thickness, overall average macular thickness and macular volume [51].

In unilateral amblyopia, we found significant difference in AL between amblyopic and fellow eyes (not only in the anisometropic subgroup), and multiple regression showed a statistically significant correlation between IOD in the thickness of most retinal layers and IOD in AL. For this reason, the effect of AL must be taken into consideration in statistical analyses to obtain reliable results, just as we did in our study. In contrast, most of the previous studies about amblyopic retinal OCT measurements were not taking into account this potential effect of AL, which could influence their results. A summary of these studies, also mentioned above, can be found in Table 6.

**Table 6:** Summary of previous studies employing optical coherence tomography of the retina in patients with amblyopia.

Study (first author, year)	Study size (n)	Age (years)	Type of amblyopia	OCT type	AL data	cpRNFL	Macular parameters(amblyopic vs fellow eyes)
**Yen (2004) ** [**21**]	38	26.4±18.3	mixed (S, A)	TD-OCT(2)	A-scan	increased	not studied
	18	25.4±18.6	A			increased	not studied
	20	27.4±18.6	S			no difference	not studied
**Yoon (2005) ** [**22**]	31	7.7 (5–12)	hyperopic A	TD-OCT(3)	ND	increased	not studied
**Altintas (2005) ** [**27**]	14	10.4 (5–18)	S	TD-OCT(3)	ND	no difference	no difference
**Kee (2006) ** [**29**]	26	8 (4–12)	mixed (S, A, AS)	TD-OCT(3)	ND	no difference	no difference
**Repka (2006) ** [**30**]	17	11.2 (5–30)	mixed (S, A, AS)	TD-OCT(3)	ND	no difference	not studied
**Huyhn (2009) ** [**25**]	48	6 and 12 year-children	mixed	TD-OCT(3)	Optical	no difference	increased FMT
**Repka (2009) ** [**31**]	37	9.2 (7–12)	mixed (S, A, AS)	TD-OCT(3)	ND	no difference	not studied
**Dickmann (2009) ** [**24**]	20	14.8 (5–47)	S (esotropia)	TD-OCT(3)	ND	no difference	increased MT and FV
	20	15.6 (6–56)	A		ND	no difference	no difference
**Walker (2011) ** [**32**]	30	56 (33–82)	mixed (S, A, AS)	SD-OCT	ND	no difference	no difference
**Pang (2011) ** [**26**]	31	9.6 (5–18)	mixed (myopic A, AS)	TD-OCT(3)	A-scan	not studied	no difference
**AL-Haddad (2011) ** [**60**]	45	20±12	mixed (S, A)	SD-OCT	ND	no difference	no difference
**Park (2011) ** [**47**]	20	9.0 (4–19)	mixed (S, A, ptosis)	SD-OCT	ND	not studied	no difference in mean FT and MT, but difference in retinal microstructure (e.g. decrease in the GCL+IPL layer)
**Dickmann (2011) ** [**23**]	15	19.7 (13–30)	S (esotropia)	TD-OCT(3)	ND	no difference	increased MT and FV
	15	19.8 (10–38)	A		ND	no difference	no difference
**Alotaibi (2011) ** [**20**]	93	8.7 (5–12)	mixed (S, A, AS)	OCT	ND	increased	no difference
	36		S			increased	no difference
	33		A			increased	increased MT and FV
	24		AS			increased	no difference
**Dickmann (2012) ** [**28**]	30	11.5 (5–23)	mixed (S, A)	SD-OCT	ND	no difference	no difference
**AL-Haddad (2013) ** [**46**]	45	20.6±13.4	mixed (S, A)	SD-OCT	ND	not studied	increased mean FT

(Abbreviations: ND: no data, FT: foveal thickness, FV: foveolar volume, MT: macular thickness, FMT: foveal minimum thikness; type of amblyopia: anisometropic amblyopia: A, strabismic amblyopia: S, combined amblyopia patients with strabismus and anisometropia: AS, TD-OCT(2): second generation time-domain OCT, TD-OCT(3): third generation time-domain OCT, SD-OCT: spectral domain OCT).

In support of our results, there is early evidence showing that photoreceptors may be affected in amblyopia. First, Enoch suggested that photoreceptor orientation is abnormal in amblyopic eyes using the Stiles-Crawford function [44], while others did not found indication of retinal dysfunction at the level of the cone photoreceptors in amblyopic eyes. Later, three groups described electro-oculographic abnormalities in amblyopic patients, with their results providing evidence for a retinal abnormality in amblyopia [54]–[56]. These results were also suggestive of the retinal pigment epithelium being involved in the process. Indeed, the RPE plays an important role in maintaining visual pigment density and perhaps also in the maintenance of photoreceptor orientation.

Leone et al. reviewed the literature on measuring macular thickness in amblyopes and proposed that increased macular thickness found by several studies may be due to inadvertent measurement of a parafoveal eccentric point in amblyopia [57]. To address this issue, central fixation was confirmed in each subject in our study by ensuring the location of the foveal depression at the center of the macular scan. Furthermore, interocular mean differences of macular thickness in non-amblyopic patients have been investigated and were minimal, even if degrees of asymmetry existed in considered individual patients.

There are several limitations to our study, namely, the small number of patients, wide age range (6–67 years), and the lack of a control group of non-amblyopic healthy patients. First, the number of patients involved in the study is comparable to those reported in most papers in the field; however, a larger study is warranted to confirm our results. Second, we were able to use the healthy eye in each patient as a control. The use of TD-OCT could limit the thickness measurements of the outermost retinal layers (e.g. the RPE) performed by OCTRIMA as compared to the improved resolution of (SD-OCT) devices. We have previously shown the high reproducibility and repeatability of OCTRIMA measurements and their comparability with SD-OCT results, therefore, we believe that our results would be similarly valid if performed by a newer generation of OCT devices [34]–[36]. Additionally, SD-OCT could detect the fine structural changes by the improved resolution and thus better segmentation of the photoreceptor outer segments. Finally, axial length could also influence the measured thickness of the retinal structures due to the reflectance directionality which can degrade the accuracy of technologies assessing RNFL thickness [58].

In addition, we would like to point out that retinal thickness measured by SD-OCT differs from that measured by TD-OCT because the delineation of the outer boundary of the retina differs in the two instruments; which corresponds to the inclusion of the outer segment–RPE–Bruch's membrane–choriocapillaris complex in the measurements. It is well known that segmentation errors are less frequent with SD-OCT. This could be attributed to the greater resolution and acquisition speed of SD-OCT. Therefore, the custom-built software used in our study facilitates the manual correction of segmentation errors by the operators; which eliminates the uncertainty of erroneous thickness calculations due to artifacts. We would like to note that a recently published study that evaluated macular thickness, agreement and intraclass repeatability in eyes with pathologies obtained by three optical coherence tomography devices (Stratus TD-OCT and two SD-OCTs, Spectralis and Cirrus OCT), revealed that although each OCT device has a unique method of defining algorithms and cannot be used interchangeably, each device has the ability to measure the retinal thickness accurately and repeatedly [59].

To the best of our knowledge, this is the first study to measure the macular thickness in unilateral amblyopia using OCT imaging and image segmentation of the entire macula area. According to our results patient age and axial length should be taken into consideration in segmentation studies in amblyopia. Using our methodology we observed subtle changes in amblyopic eyes affecting the outer nuclear layer of the fovea suggesting the possible involvement of the photoreceptors. However, further studies are warranted to support this hypothesis.
